# A Brighter Outlook on the Hospital's Position

**Published:** 1920-09-11

**Authors:** 


### THE LONDON HOSPITAL.

### A Brighter Outlook on the Hospital's Position.

At the quarterly court of governors held on September 1 it was announced that the committee have considered the following propositions to meet the financial crisis:—(1) Closing the entire hospital; (2) closing a wing of 300 beds; (3) converting some of the wards into paying wards at a charge of six guineas a week.

As regards the first proposal—the closing of the entire hospital—the prospect of the distress of the sick poor which such a step would entail is certainly appalling; in addition, there has to be considered the throwing out of employment of some hundreds of nurses and others, and the dissolution of an organisation which has been the growth of years.

The second proposal—the closing of a wing—would not save the situation, as many establishment charges would remain whether the beds were 600 or 900. It is estimated that to close a wing temporarily would save £10,000 a year, or permanently £20,000. Permanent closing would, of course, include the dismissal of a proportionate number of employees.

As regards converting part of the hospital into paying wards at a charge of six guineas a week, although many people would gladly avail themselves of these terms, it was felt that to cater for this class was not the object for which the hospital in a poor district of London was founded.

Ultimately it was decided to carry on the work of the hospital without curtailment until funds were exhausted, and to inform the Ministry of Health and King Edward's Hospital Fund of the desperate financial position.

A letter was sent to the King's Fund on June 17, 1920, pointing out that as far as could be foreseen the hospital could continue its work until the end of August, and would then, unless special assistance was forthcoming, close. In addition to warning the Ministry of Health and the King's Fund, Lord Knutsford informed the public in an appeal letter to the press. This was no mere cry of wolf; hard facts and figures had to be faced. All available securities had been pledged to the bank, and the bankers would lend no more.

King Edward's Hospital Fund made a special donation of £35,000, and there was a good response to Lord Knutsford's appeal—the total amount received to date being over £50,000. It is customary for the hospital to appeal widely only every five years, and the last quinquennial appeal was no farther distant than 1918; but in the crisis which has arisen it was felt that the hospital should not close its gates without giving the public the opportunity of coming to the rescue. The appeal was a success, not only financially as tiding over the immediate difficulties, but also as showing that charity is not dead, though it may be unable, on account of the enormously increased cost of running the hospital, to bear the entire burden as heretofore.

In future, three sources for the maintenance of the hospital must be looked to, namely:—(1) Voluntary contributions; (2) payment by patients of a proportion of the cost of maintenance; (3) State assistance.

### Payment by Patients.

The governors decided at the March court to put into force a system of payment. It took some time to work out the details, but the scheme has been in operation long enough to show that the innovation adopted has been clearly justified. The following are the principles of the scheme:—

All adult in-patients are required to pay 3s. a day—£1 1s. a week—towards the cost of their maintenance. Two weeks' payment is demanded in advance, but a proportionate refund is made if the patient does not stay in hospital for the whole time for which he has made advance payment.

The total cost of each in-patient is £4 4s. 6d. week. The charge of £1 1s. was adopted as being a reasonable proportion for the individual to bear, and at the same time, as having some relation to the amount of an individual's food and maintenance if he were well and at home. It is also considered to be an amount which is within the means of the artisan and mechanic class, who are the chief users of the hospital.

A good deal of discussion took place as to whether a flat rate should be charged, or whether each patient should be assessed according to means. The committee ultimately adopted the flat rate. It was the opinion that to assess each patient would necessitate a large and expensive staff, and the work would be particularly difficult in the case of the London Hospital which, owing partly to the facilities for special forms of treatment unobtainable elsewhere, draws its patients from a wide and scattered area.

From the patients' point of view a flat rate is more satisfactory. The individual knows definitely the amount which will be required if he falls sick and needs hospital treatment—this will be more especially the case if a flat rate charge becomes general among hospitals. A person by joining a benefit club can more readily make provision against a definite charge for hospital treatment than against an indefinite amount to be fixed at the arbitration of an assessor.

Hitherto, the working man in his budget has relied on free hospital treatment—his wages did not permit it to be otherwise. Now, as far as the "London" is concerned, hospital treatment will cost a guinea a week. There are indications that other hospitals will follow the "London" in this respect.

Children under seven are treated free, between seven and fourteen at half rate. Seven years is the age-limit of the baby wards. It may be argued that since a child of seven or under is not a wage-earner the parents lose nothing when the child is sick, and would be none the worse off if they paid towards

**The London Hospital**—(*continued*).

the child's maintenance when in hospital. On the other hand, it is realised that every encouragement ought to be given to parents to see that their children are well looked after medically, and it was thought that to make a charge for children under seven might tend to delay action on the part of the mother until the baby "got much worse."

Accident and emergency cases are, of course, admitted without any question, but arrangements are made, when possible, with friends or relatives for payment.

Although the £1 1s. a week for adults is the definite charge, exemptions are made. For instance, a patient who needs a surgical operation or some special treatment which cannot be obtained at an infirmary will not be refused admission solely on account of inability to pay. So far the statistics show that ten per cent. of the patients are exempted. The average takings reach about £50 a day, so it is reasonable to estimate that from £15,000 to £20,000 a year will accrue to the hospital from patients' payments.

It is possible that with payment in force voluntary contributions will diminish; at present it is too early to give any definite opinion on this point, but there are indications that there will not be any serious diminution. The committee, however, wish to emphasise that voluntary contributions plus payment by patients are necessary if the hospital is to keep open.

Quite the most gratifying feature is the willingness on the part of the majority of patients to pay. They realise the necessity and acknowledge the justness of the charge.

Government Aid.

As regards aid from the Ministry of Health, so far no definite proposition has been put forward. Whatever form it may take, the committee are strongly of the opinion that payment should be made towards the cost of the treatment of insured persons in hospital. As matters are at present, the panel doctor is paid for the treatment of the more minor ailments, but for any serious illness requiring nursing and special medical or surgical skill, the patient has to be sent to the hospital, and the hospital gets nothing.

In any event a definite policy is badly needed, so that hospitals may know how much of the bill will be paid by the State, how much will be required from the individual, and how much from voluntary contributions.

